# A study on the relationship between work stress and turnover intentions among critical care nurses: mediating roles of job satisfaction and burnout

**DOI:** 10.3389/fpubh.2026.1744177

**Published:** 2026-01-21

**Authors:** Yunfan Ji, Wei Hu, Di Xu, Fengzhi Chai, Yuhong Wang, Caiyue Xu, Xia Li

**Affiliations:** 1 School of Nursing, Jinzhou Medical University, Jinzhou, Liaoning, China; 2 First Affiliated Hospital of Jinzhou Medical University, Jinzhou, China

**Keywords:** job satisfaction, job stress, occupational burnout, parallel mediation, turnover intention

## Abstract

**Background:**

Nursing staff shortages and the loss of nursing talent resources remain persistent global challenges. Work stress, job satisfaction, and occupational burnout are key factors influencing nursing staff turnover intentions. Examining the interrelationships among these four variables can provide deeper insights into turnover issues among critical care nurses.

**Objective:**

To examine the relationship between work stress and turnover intention among nurses in intensive care units. Additionally, this study aims to explore the mediating roles of occupational burnout and job satisfaction, thereby investigating the underlying mechanisms linking work stress, job satisfaction, occupational burnout, and turnover intention within the nursing population.

**Method:**

A cross-sectional survey was conducted among 257 intensive care unit nurses. Study variables included job stress, job satisfaction, occupational burnout, and turnover intention. Key data underwent bivariate Spearman correlation analysis and mediation analysis using SPSS 27.0 and R 4.4.2.

**Results:**

Job stress was negatively correlated with job satisfaction (*r* = −0.704, *p* < 0.01) and positively correlated with burnout (*r* = 0.616, *p* < 0.01) and turnover intention (*r* = 0.758, *p* < 0.01). Job satisfaction significantly negatively influenced turnover intention (*r* = −0.742, *p* < 0.01), while occupational burnout significantly positively influenced turnover intention (*r* = 0.663, *p* < 0.01). The structural equation model demonstrated good fit (*χ*^2^/*df* = 2.55; CFI = 0.93, TLI = 0.92; RMSEA = 0.077). Job stress significantly and directly influenced turnover intention (*β* = 0.23, *p* = 0.003), while job satisfaction (*β* = 0.098, 95% CI: 0.046–0.153) and occupational burnout (*β* = 0.083, 95% CI: 0.045–0.132) concurrently mediated the relationship between job stress and turnover intention. In the overall effect of work pressure on turnover intention, indirect effects (through job satisfaction and burnout) collectively account for 56.56%, while direct effects account for 43.44%.

**Conclusion:**

This study examined the relationship between job stress and turnover intention, as well as the direct and indirect effects of enhancing job satisfaction and reducing occupational burnout on lowering nurses’ turnover intention. It provides theoretical foundations and practical implications for mitigating nurse turnover issues.

## Introduction

The accelerating global population aging and rising healthcare demands have placed nurses under increasing work pressure. Nursing is considered a high-risk profession due to the daily exposure to challenging situations such as death and pain management (1). In particular, ICUs may face pressures due to high mortality rates, critically ill patients, and ethical dilemmas (2). When staff lack sufficient time to provide adequate care for each patient, this situation may worsen (3). Additionally, high levels of dissatisfaction among nurses also contribute to staff turnover in the nursing profession (4). According to statistics from the National Health and Family Planning Commission, the average turnover rate for nurses is 8.2% (5). High turnover rates among nurses can adversely affect patient care quality and the overall functioning of the healthcare system (6).

Data for this study were collected from three Grade III Class A hospitals in Jinzhou City, Liaoning Province, China, and one Grade III Class A hospital in Shangrao City, Jiangxi Province. As regional medical centers, these hospitals collectively operate 120 ICU beds and admit over 5,000 critically ill patients annually. China’s ICU nursing system is characterized by “high patient volume, high technical dependency, relatively strained nursing resources (average nurse-to-patient ratio of 1:2.5), and widespread shift work (primarily three-shift rotations).” These contextual factors may collectively exacerbate nursing workload stress and increase turnover risk. As a high-pressure environment within the healthcare system, the unique demands of nursing work in intensive care units further amplify stress levels and the risk of staff turnover. From a physiological perspective, rotating day and night shifts disrupt nurses’ circadian rhythms, increasing the risk of sleep disorders and metabolic syndrome. Psychologically, frequent exposure to life-and-death situations and managing critical conditions can lead to emotional exhaustion among nurses. Compounded by limited career advancement opportunities, ICU nurses commonly experience high levels of emotional fatigue and low personal fulfillment (7).

A 2018 study revealed that intensive care unit (ICU) nurses face a higher risk of burnout: 11% of ICU nurses exhibited high emotional exhaustion risk, and 7% planned to leave the nursing profession (8). The loss of experienced nurses, particularly specialized nurses such as those in intensive care units, adversely affects the provision and continuity of nursing services and may lead to higher rates of nursing errors and patient mortality (9). Frequent staff turnover leads to gaps in nursing services, increased rates of nursing errors, and heightened workloads for retained nurses, creating a vicious cycle (10).

The persistently high turnover rate among ICU nurses worldwide has become a critical issue affecting the quality of critical care. Multiple systematic reviews and meta-analyses in recent years have confirmed that work stress, job satisfaction, and professional burnout are core variables influencing ICU nurses’ turnover intentions (11). However, significant research gaps remain. First, previous ICU nurse studies have predominantly focused on single mediating variables, failing to simultaneously examine the independent effects and relative importance of multiple pathways. For instance, Al Sabei et al. (12) surveyed 207 nurses in Omani public hospitals and found job satisfaction moderates the relationship between burnout and turnover intention. However, they did not treat satisfaction as a parallel mediator alongside burnout, overlooking its potential to independently transmit stress effects. Similarly, Quesada-Puga et al. (13) conducted a meta-analysis of 18 studies, revealing that both job satisfaction and burnout are closely associated with turnover intention. However, the “dual parallel mediation mechanism” through which work stress influences turnover intention remains unclear. Second, research in the specialized field of ICU nursing remains insufficient. For instance: Lai et al. (14) meta-analysis revealed that job stress indirectly influences turnover intention through occupational burnout, yet ICU nurses constituted less than 10% of their study sample. Additionally, Poon et al. (15) conducted a systematic review of healthcare worker turnover intentions during the COVID-19 pandemic, examining turnover intentions among healthcare personnel but not specifically targeting the specialized ICU nursing population. Finally, large-scale studies (*n* ≥ 200) focusing on Chinese ICU nurses remain scarce. Ramírez-Elvira et al. (7) examined the prevalence, risk factors, and burnout levels among intensive care unit nurses: Only one Chinese study was included in the systematic review and meta-analysis, and the model was not validated against the domestic context of high nurse-to-patient ratios and intensive night shifts, limiting the generalizability of the conclusions. This study addresses these gaps by constructing a parallel mediation model, integrating characteristics of the Chinese ICU nursing population, and applying theoretical guidance. It will elucidate the intrinsic pathway through which ICU nurses’ work stress translates into turnover intentions and provide evidence-based foundations for developing evidence-based interventions—a critical measure not only for safeguarding ICU nurses’ well-being but also for meeting the urgent global need to enhance critical care quality.

### Intention to leave

Intention to leave refers to an individual’s thoughts and inclination to resign from their current job, serving as a core indicator for predicting actual turnover behavior. In this study, intention to leave specifically refers to nurses’ desire to depart from their current ICU position and the hospital where they are employed, and does not include the tendency to leave the nursing profession. The global shortage of health professionals is severe, with the current gap reaching 7.2 million and projected to double in the coming decades (16). Factors contributing to nurses’ intention to leave include occupational burnout, insufficient organizational commitment, low job satisfaction, and deficiencies in human resource management (17). International research indicates that 41% of hospital nurses in countries such as the United States, Canada, and England are dissatisfied with their jobs, with 22% planning to leave within 1 year (18); Data from Asian countries is equally concerning: the proportion of primary healthcare workers dropped from 44% to 33% between 2010 and 2016 (19). The intention to leave among ICU nurses ranges from 11.0% to 76.3% (20–22), with turnover rates for newly hired ICU nurses reaching approximately 30% within 3 months and 57.7% within 1 year (23).

### Work pressure

Work stress is an individual’s psychological and physiological response to an imbalance between demands and resources in the work environment. When job requirements exceed personal capabilities or available resources, it triggers the experience of stress. Stress may also serve as an indicator of overload, conflict, and ambiguity arising from the work environment (including personal characteristics) (24). Previous studies have confirmed that work stress is directly related to nurses’ intention to leave their jobs (25), meaning that the greater the stress, the higher the likelihood of nurses resigning. However, the relationship between the two is more complex in the ICU setting: However, within the intensive care unit environment, the relationship between the two becomes more complex: First, the workload is overwhelming, requiring 24-h monitoring of patient conditions and performing high-risk procedures; Second, the psychological burden is significant, as frequent exposure to patient suffering and death can lead to empathy fatigue; Third, inadequate environmental support exacerbates stress through issues such as staffing shortages, communication barriers, and lack of management support. This pressure influences resignation decisions through more intense psychological impact. Although some studies have explored moderating variables such as organizational commitment and social support (26), the specific pathways through which ICU nurses’ work stress translates into turnover intentions remain unclear.

### Job satisfaction

Job satisfaction reflects an individual’s emotional experience and level of fulfillment regarding their work content and environment. It is influenced by multiple factors, such as the nature of the work tasks themselves, relationships with colleagues, and organizational-level support (27, 28). Given the ICU’s overwhelming workload and high-risk environment, it is hardly surprising that critical care nurses experience dissatisfaction and burnout. When nurses remain in a state of “high investment with low returns” for extended periods, unable to derive a sense of accomplishment or security from their work, their satisfaction levels decline significantly, ultimately fostering thoughts of leaving. A study indicates that work stress has a detrimental effect on job satisfaction (13). Another study indicates that nurses’ job satisfaction is significantly negatively correlated with their intention to resign (29). Based on JD-R theory (Job Demands-Resources Theory), work stress fundamentally stems from an imbalance between “job demands and job resources,” while job satisfaction represents the direct psychological outcome of this imbalance: greater stress leads to more negative evaluations of work and lower satisfaction. Simultaneously, job satisfaction serves as a key predictor of turnover intention, with declining satisfaction directly intensifying the desire to leave. Based on this, it is hypothesized that work stress indirectly increases nurses’ turnover intention by reducing their job satisfaction, meaning job satisfaction mediates the relationship between the two variables. This hypothesis is supported by existing research: Ho et al. (30) found a significant negative correlation between job stress and nurses’ job satisfaction. Another study showed that a one-point increase in job satisfaction reduces nurses’ turnover intention by 71% (12), with job satisfaction mediating 38.86% of the relationship between stress and turnover intention.

### Burnout

Burnout is defined as a psychological state primarily caused by work-related stressors. The World Health Organization defines burnout as “a syndrome resulting from chronic workplace stress that has not been successfully managed” (30). Burnout is a state of depleted personal resources resulting from chronic work stress, characterized by intense emotional exhaustion, a lack of personal accomplishment, and depersonalization (31). Nurses constitute a high-risk group for occupational burnout, with an overall incidence rate of 28% (32). Frontline nurses face significantly higher burnout risks than general ward nurses due to greater stress intensity. Specifically, 60.5% of ICU nurses exhibit moderate to high levels of emotional exhaustion, 42.3% demonstrate depersonalization, and 60.6% experience depersonalization (33). Professional burnout serves as the critical bridge connecting work stress to resignation intent: Prolonged high stress gradually depletes ICU nurses’ psychological resources, triggering emotional exhaustion, which in turn leads to depersonalization (indifference toward patients) and diminished personal accomplishment (perceiving work as valueless). This state of burnout directly fuels resignation intent: Emotional exhaustion leaves nurses unable to continue working, depersonalization erodes their professional identity, and diminished personal accomplishment reduces their willingness to stay. Lin et al. (34) demonstrated that occupational burnout significantly increases employee absenteeism and job-hopping intentions, particularly in high-stress settings like intensive care units where burnout more strongly predicts turnover intentions. This suggests that work stress indirectly elevates turnover intentions among critical care nurses by exacerbating their occupational burnout, indicating that burnout mediates the relationship between the two.

### Theoretical foundations

#### Core theoretical framework: job demands-resources (JD-R) theory

The Job Demands-Resources (JD-R) Theory, proposed by Bakker and Demerouti (35), was selected as the core guiding theory for this study, based on its direct alignment with our research variables, contextual fit with ICU nursing, and strong empirical support in nursing literature. The theory influences turnover intention through two independent pathways: First, by depleting psychological resources to induce occupational burnout (health impairment pathway); second, by diminishing perceived job rewards to lower job satisfaction (the motivation pathway). For ICU nurses, “high demands” include heavy workloads (24-h critical patient monitoring, high-risk procedures), frequent exposure to death, and ethical dilemmas; “low resources” involve staffing shortages, limited psychological support, and restricted career development. This imbalance directly induces physical/mental exhaustion, reduces job satisfaction, and elevates occupational burnout—ultimately increasing turnover intention. This relationship chain (work stress → satisfaction/burnout → turnover intention) is exactly the mechanism our study aims to validate, making the JD-R Theory the most logical core framework.

### Supplementary theoretical context

Two classic organizational behavior theories are briefly introduced to contextualize the JD-R framework, rather than serving as independent guiding models:

*Maslow’s hierarchy of needs theory*: This theory clarifies how ICU work stress disrupts the “demands-resources balance” in the JD-R model by undermining multi-level need fulfillment (36). For example, shift work disrupts physiological needs (sleep deprivation), high-risk procedures threaten safety needs (occupational anxiety), and limited career development restricts self-actualization needs. Unmet needs accumulate as negative psychological resources, exacerbating the “health impairment pathway” of the JD-R Theory.
*Herzberg’s two-factor theory*: This theory explains why high ICU demands reduce job satisfaction (a key mediator in our model) (37). ICU stressors (e.g., excessive workloads, irregular shifts) erode “hygiene factors” (working conditions, work-life balance), directly triggering dissatisfaction; meanwhile, emotional exhaustion from stress prevents nurses from gaining “motivators” (sense of accomplishment from patient recovery, professional recognition), further lowering satisfaction. This aligns with the JD-R Theory’s proposition that low resources diminish positive psychological outcomes (job satisfaction).

### Theoretical propositions and model construction

Based on JD-R theory, we propose Theoretical Proposition: Job stress (high job demands) influences occupational burnout (health impairment), which in turn affects turnover intention; Job stress (high job demands/low job resources) correlates with job satisfaction (reduced motivation), thereby influencing turnover intention;

Based on Maslow’s Hierarchy of Needs theory, we propose Theoretical Proposition: Work stress influences both job satisfaction and burnout by affecting multiple levels of needs—physiological, safety, and esteem—thereby jointly impacting turnover intention;

Based on Herzberg’s Two-Factor Theory, Theoretical Proposition proposes: Work stress impacts job satisfaction by undermining hygiene factors (e.g., work environment, compensation) and triggers burnout by depleting psychological resources, with both pathways concurrently influencing turnover intention.

### Link to research gap

While the JD-R Theory has been used to explore stress and turnover in general nursing settings, it has not yet been applied to examine the parallel mediating roles of job satisfaction and occupational burnout in ICU nurses—a group facing unique “life-responsibility stress” (e.g., procedural errors leading to patient death) that amplifies the “demands-resources imbalance.” Maslow’s and Herzberg’s theories help contextualize this uniqueness but do not replace the JD-R Theory as the core framework. Our study fills this gap by testing a JD-R-based model to clarify how ICU nurses’ work stress translates into turnover intention via dal mediators.

Research objectives:

1. To validate the direct relationship between ICU nurses’ work stress and turnover intention.
2. To explore the parallel mediating roles of job satisfaction and occupational burnout in the aforementioned relationship.

Research hypotheses:

*H1*: ICU nurses’ work stress is positively correlated with turnover intention.

*H2*: ICU nurses’ work stress is negatively correlated with job satisfaction and positively correlated with occupational burnout.

*H3*: Job satisfaction among ICU nurses is negatively correlated with turnover intention, while occupational burnout is positively correlated with turnover intention.

*H4*: Job satisfaction mediates the relationship between ICU nurses’ job stress and turnover intention.

*H5*: Burnout mediates the relationship between ICU nurses’ job stress and turnover intention.

*H6*: Job satisfaction and burnout jointly mediate the relationship between ICU nurses’ job stress and turnover intention.

**Figure 1:**
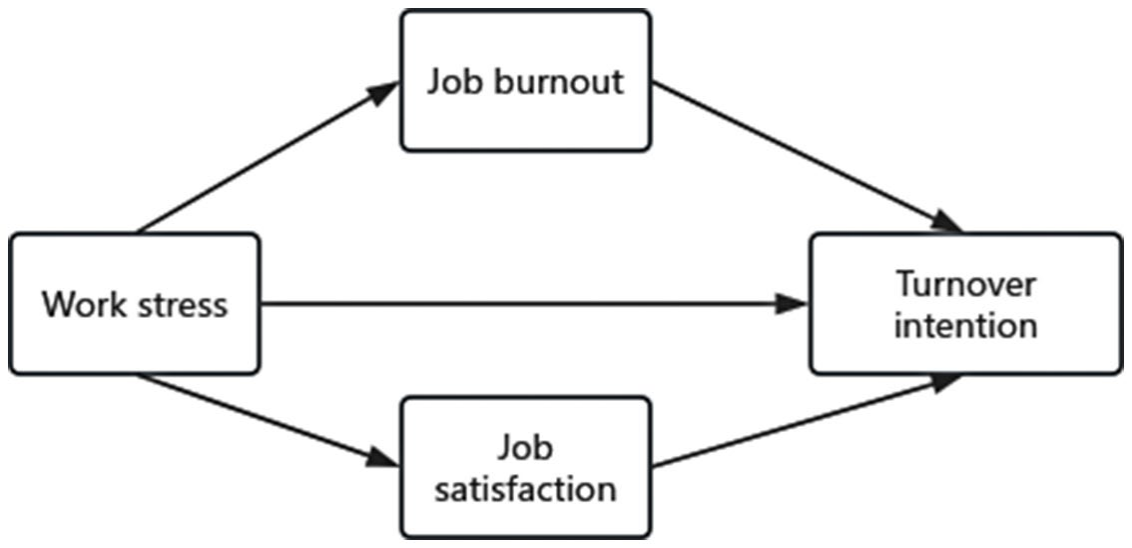
This theoretical model diagram is based on the aforementioned theory and the relationships among the four variables. It illustrates the direct relationship between work pressure and turnover intention, as well as the parallel mediating relationship between job satisfaction and occupational burnout.

Based on the aforementioned theoretical and variable relationship analysis, the theoretical model proposed in this study is shown in Figure 1.

## Method

### Participants

This study employed a cross-sectional design, collecting data via paper-based questionnaires. Convenience sampling was utilized, primarily based on sample accessibility. The specific recruitment process is as follows: Collaborative Coordination: In September 2024, the research team collaborated with nursing departments of four Grade A tertiary general hospitals in Liaoning and Jiangxi provinces to clarify research objectives, data confidentiality principles, and recruitment requirements. Department Briefings: ICU head nurses at each hospital provided detailed explanations to department nurses regarding the study content, participant rights, and questionnaire completion guidelines to ensure informed consent. Voluntary Recruitment: Nurses meeting inclusion criteria voluntarily participated in the study. Questionnaire Distribution and Collection: Researchers visited hospitals weekly for on-site distribution and collection, excluding ineligible participants such as interns and residents. Data Screening: This study strictly adheres to the core standard from Kline (38), Principles and Practice of Structural Equation Modeling: SEM models of moderate complexity require at least 200 samples. Given the unique nature of the ICU nursing population, strict inclusion criteria and recruitment challenges necessitated collaboration across four hospitals. A total of 280 questionnaires were distributed, yielding 257 valid responses (response rate: 91.8%). This sample size represents the maximum feasible scale within the accessible population and meets the minimum sample size requirement for structural equation modeling. The data collection period spanned from October 2024 to January 2025.

Inclusion and exclusion criteria:

*Inclusion criteria*: Registered nurses working in ICUs at multiple tertiary hospitals within China; At least 6 months of work experience; Voluntary participation in the study and signing of informed consent. Exclusion Criteria: Nurses on long-term sick leave or maternity leave; Nurses planning to transfer out of the ICU or resign during the study period; Nurses visiting the hospital for training or residency programs.
*Adaptive note*: The core objective of this study is to validate the associative patterns and mediating effects among variables, rather than inferring strict causal relationships. The cross-sectional design can preliminarily reveal the potential relationships between variables through correlation analysis and structural equation modeling, laying the groundwork for subsequent longitudinal research.

### Measures

Demographic and practice characteristics questionnaire: Designed by the researchers, covering: Demographic characteristics: gender, age, marital status, professional title, educational attainment, monthly salary, years of service, employment status; Practice characteristics: department, hospital type, ICU bed capacity, number of patients admitted to the department, department patient mortality rate, number of nurses in the department, patient-to-nurse ratio in the department.

*Work stress*: The Nursing Stress Scale (NSS), introduced and revised by Li and Liu (39), is used to measure nurses’ work stress levels (40). The scale comprises five dimensions (nursing professionalism and work issues, time allocation and workload issues, work environment and equipment issues, patient care issues, management and interpersonal relationship issues), totaling 35 items. The Likert 4-point scale (1 = no stress, 4 = severe stress) was employed, with higher total scores indicating greater stress levels. The Cronbach’s *α* coefficient for the total scale was 0.98, while the Cronbach’s *α* coefficients for each dimension ranged from 0.83 to 0.95 (39), The Cronbach’s alpha coefficient for the nursing workload scale in this study was 0.88, demonstrating good reliability and validity.

*Job burnout*: The Maslach Burnout Inventory-Discriminative Dimension (MBI-DS) is currently the most widely used and accepted measurement tool for job burnout, having demonstrated excellent reliability and validity (42). This study employs the Chinese version of the Maslach Burnout Inventory—Human Services Survey (MBI-HSS), validated and licensed from Mind Garden, Inc. The scale comprises three dimensions (emotional exhaustion, depersonalization, and reduced personal accomplishment) and employs a 7-point Likert scale. Cronbach’s alpha coefficients across dimensions ranged from 0.82 to 0.88 [Li and Li, (45)], The Cronbach’s alpha coefficients for each dimension of the occupational burnout scale in this study ranged from 0.76 to 0.87, meeting the requirements for reliability and validity in the research.

*Job Satisfaction*: The McCloskey/Mueller Satisfaction Scale (MMSS) (43) is a multidimensional measurement tool designed to assess job satisfaction among professional nurses. The survey comprises 8 dimensions (colleague relationships, job control and responsibility, work-family balance, benefits, scheduling, etc.) with 31 items in total. It employs a 5-point Likert scale (1 = Very dissatisfied, 5 = Very satisfied). An average score ≥3.03 indicates meeting the job satisfaction benchmark, with higher total scores reflecting greater satisfaction (44). The Chinese version of the total scale achieved a Cronbach’s *α* coefficient of 0.95, with individual dimension coefficients ranging from 0.60 to 0.84, indicating good reliability and validity. The Cronbach’s alpha coefficient for the job satisfaction scale in this study was 0.911.

Intention to leave: The Nurse Intention to Leave Scale (TIS), translated and revised by Li and Li (45) measures nurses’ intention to leave their current positions. The scale comprises three dimensions (likelihood of resigning from current job, motivation to seek alternative employment, likelihood of obtaining external employment) and consists of six items. The Likert 4-point scale (1 = Never, 4 = Very Often) yields a total score ranging from 6 to 24 points, with higher scores indicating stronger turnover intention. The Cronbach’s alpha coefficient for the Nurse Turnover Intention Scale in this study was 0.897.

### Data analysis

Research data were analyzed using SPSS 27.0 and R 4.4.2 software. Statistical analysis was performed via SPSS: participants’ demographic characteristics were presented through descriptive analysis, with categorical variables expressed as frequencies and percentages, and continuous variables represented by means and standard deviations. Variable distributions were examined using skewness and kurtosis with corresponding critical values. Spearman correlation analysis was performed using SPSS 27.0 to test for zero-order correlations among variables, while internal consistency reliability was measured via Cronbach’s alpha. Perform structural equation modeling analysis using the lavaan package in R 4.4.2, while simultaneously conducting mediation effect tests. Before constructing the structural model, conduct confirmatory factor analysis (CFA), Model fit was evaluated using *R* mediation analysis, with specific metrics as follows: Chi-square (*χ*^2^), degrees of freedom (*df*), *χ*^2^/*df* ratio, root mean square error of approximation (RMSEA), Tucker-Lewis index (TLI), standardized root mean square residual (SRMR), comparative fit index (CFI), normality fit index (NFI), and good fit index (GFI). All path coefficients (associations between study variables) are presented. Indirect effects were calculated using the bias-corrected bootstrap method (5,000 bootstrap samples). Bilateral tests yielded *p* < 0.05, indicating statistically significant differences.

## Results

### Distribution of demographic characteristics and work context variables

Demographic characteristics of the 257 participants are shown in Table 1. Distribution across general and specialty ICUs: General ICU nurses accounted for 66.9%, neonatal ICU for 14.4%, emergency ICU for 7.4%, respiratory ICU for 8.6%, and surgical ICU for 2.7%. Nurse-to-patient ratio: Day shift average: 1:2 Night shift average: 1:2.6 Workload indicators: Average weekly nursing hours: 40 h Monthly night shift frequency: 8 shifts. The majority (143, 55.6%) were aged 20–30 years. Gender distribution: 47 males (18.3%) and 210 females (81.7%). Marital status: 136 married (52.9%) and 119 unmarried (46.3%). Educational attainment: Undergraduate degree holders (170, 66.1%) constituted the majority. Monthly income: 114 participants (44.4%) earned between ¥5,001–8,000, while 47 (18.3%) earned over ¥8,000. Monthly days off: 181 participants (70.4%) had 5–8 days off per month. Additional general information is presented in Table 1.

**Table 1:** Distribution of demographic characteristics and work context variables of the study participants (*n* = 257).

Demographic	Sample (*n* = 257)	Frequency	Percentage	Mean ± SD
Average weekly working hours				40
Monthly night shifts				8.00
Nurse-to-patient ratio	Nurse-to-patient ratio-day			2.00
	Nurse-to-patient ratio-night			2.60
Department	GICU	172	66.9%	
	NICU	37	14.4%	
	EICU	19	7.4%	
	RICU	22	8.6%	
	SICU	7	2.7%	
Age	20–30 year	143	55.6%	
	31–40 year	104	40.5%	
	≥41 year	10	3.9%	
Gender	Male	47	18.3%	
	Female	210	81.7%	
Marital status	Unmarried	119	46.3%	
	Married	136	52.9%	
	Cohabiting	1	0.4%	
	Divorced	1	0.4%	
Education	Secondary specialized school	5	1.9%	
	Junior college	76	29.6%	
	Undergraduate	170	66.1%	
	Master’s degree	6	2.3%	
Children	No children	143	55.6%	
	Have children	114	44.4%	
Monthly income	Below 3,000 yuan	14	5.4%	
	3,001–5,000 yuan	82	31.9%	
	5,001–8,000 yuan	114	44.4%	
	8,001–12,000 yuan	46	17.9%	
	12,001–15,000 yuan	1	0.4%	
Monthly rest days	0–4 day	25	9.7%	
	5–8 day	181	70.4%	
	9–12 day	35	13.6%	
	13–16 day	15	5.8%	
	>17 day	1	0.4%	
Shift pattern	8 h	179	69.6%	
	12 h	61	23.7%	
	16 h	10	3.9%	
	24 h	7	2.7%	
Schedule satisfaction	Extremely dissatisfied	5	1.9%	
	Relatively dissatisfied	18	7.0%	
	Average	119	46.3%	
	Relatively satisfied	65	25.3%	
	Extremely satisfied	50	19.5%	

### Variable correlations

Spearman’s correlation analysis was employed to examine the relationships among work stress (NSS), job satisfaction (MMSS), burnout (EE, DP, PA), and nurses’ turnover intention (TIS). Results indicated that work stress showed a significant negative correlation with job satisfaction (*r* = −0.704, *p* < 0.01), significantly positively correlated with all burnout dimensions (EE, DP, PA) (0.604, 0.606, 0.379, all <0.01), and significantly positively correlated with turnover intention (*r* = 0.758, *p* < 0.01); Job satisfaction showed a significant negative correlation with turnover intention (*r* = −0.742, *p* < 0.01). Each dimension of burnout exhibited a significant positive correlation with nurses’ turnover intention (0.629, 0.579, and 0.474, respectively, all *p* < 0.01). All correlations between variables were statistically significant at the 0.01 level (two-tailed). See Table 2 for specific data: Spearman correlation coefficients between variables.

**Table 2:** Discriminant validity.

Variable	NSS	TIS	MMSS	EE	DP	PA
NSS	1					
TIS	0.758	1				
MMSS	−0.704	−0.742	1			
EE	0.604	0.629	−0.580	1		
DP	0.606	0.579	−0.513	0.777	1	
PA	0.379	0.474	−0.318	0.481	0.456	1

### Mediation analysis

*Direct effect results*: Job stress significantly and negatively predicted job satisfaction (*β* = −0.38, *p* = 0.001); job satisfaction significantly and negatively predicted nurses’ turnover intention (*β* = −0.098, *p* = 0.001); Job stress significantly and positively predicts occupational burnout (*β* = 1.87, *p* = 0.001); occupational burnout significantly and positively predicts nurses’ turnover intention (*β* = 0.083, *p* = 0.000); job stress exerts a significant positive direct effect on nurses’ turnover intention (*β* = 0.23, *p* = 0.003).

*Indirect effect results*: The indirect effect value of work pressure on nurses’ turnover intention via job satisfaction was 0.098, with a 95% confidence interval of [0.046, 0.153], which did not include 0 and was statistically significant; The indirect effect value of job stress on nurses’ turnover intention via occupational burnout was 0.083, with a 95% confidence interval of [0.045, 0.132], which did not include zero and was statistically significant; The total indirect effect of work pressure on nurses’ intention to leave via job satisfaction and occupational burnout was 0.181, with a 95% confidence interval of [0.106, 0.261], which did not include zero and was statistically significant.

*Total effect and contribution percentages*: Job satisfaction accounted for 30.51% of the total effect through its mediating role, while occupational burnout contributed 26.06%. Together, these mediating effects accounted for 56.56% of the total effect, with the direct effect comprising the remaining 43.44%. The *R*^2^ value in this study is 0.747, indicating that the model explains 74.7% of the variance in turnover intention.

**Figure 2:**
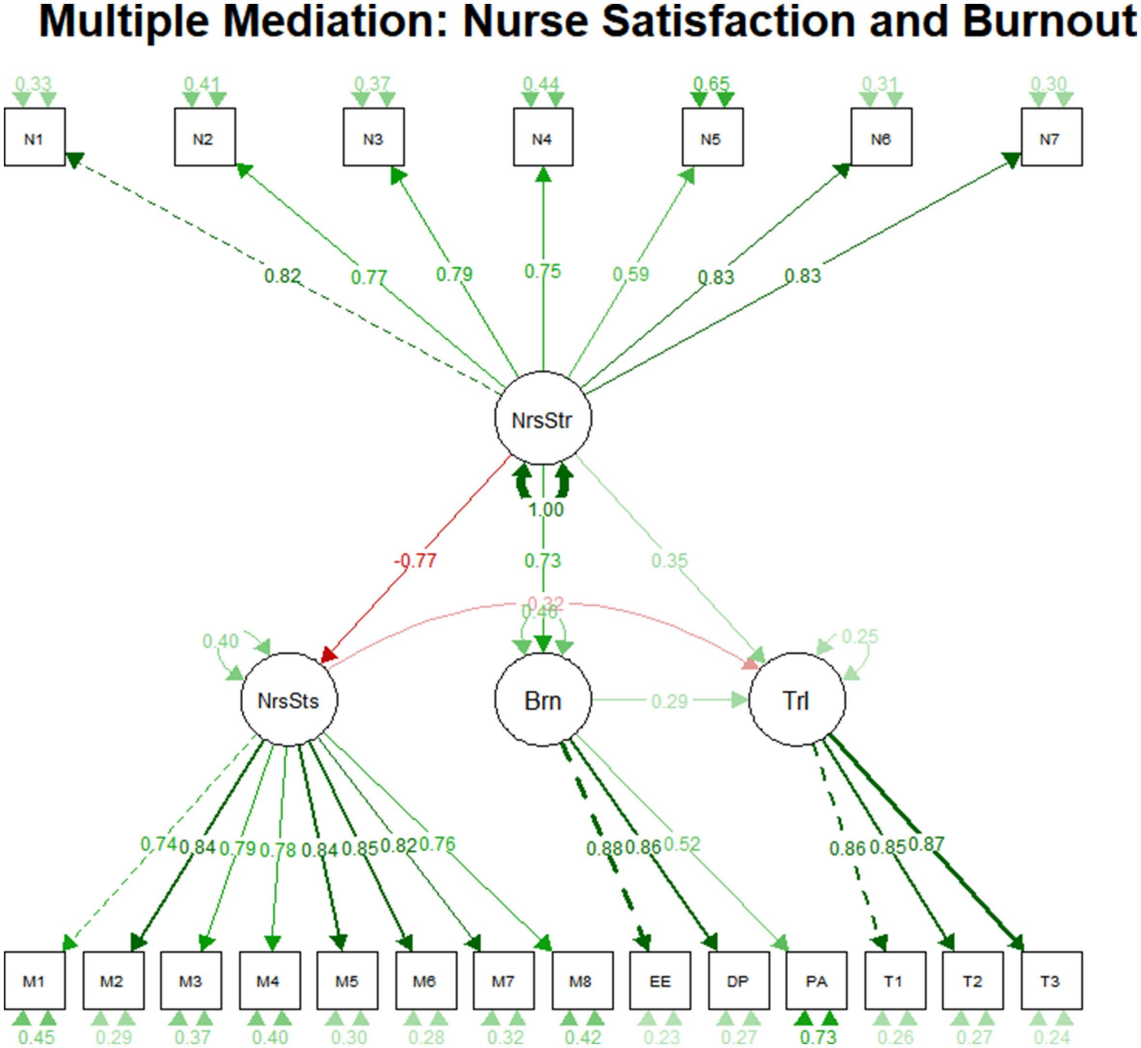
Final relationship diagram and path coefficient map among the four variables.

Detailed data are shown in Table 3: Direct effects, indirect effects, and total effects of the hypothetical model. The final model path relationship diagram is shown in Figure 2.

**Table 3:** Path coefficients, standard errors, and significance test results of the structural equation model.

Path label	Est	Se	*p* value	CI.lower	CI.upper	Significance
Direct effect						
Nurse satisfaction → nurse stress	−0.466	0.042	0.000	−0.551	−0.380	***
Turnover intention → nurse satisfaction	−0.210	0.061	0.001	−0.325	−0.098	***
Burnout → nurse stress	1.610	0.129	0.000	1.376	1.875	***
Turnover intention → burnout	0.052	0.014	0.000	0.028	0.083	***
Turnover intention → nurse stress	0.139	0.046	0.003	0.050	0.235	**
Indirect effect						
Indirect1	0.098	0.028	0.000	0.046	0.153	***
Indirect2	0.083	0.023	0.000	0.045	0.132	***
Indirect total	0.181	0.039	0.000	0.106	0.261	***
Total effect						
Total	0.32	0.026	0.000	0.276	0.376	***

*Model fit indices*: The core fit indices of this study’s model (*χ*^2^/*df* = 2.55, CFI = 0.93, TLI = 0.92, RMSEA = 0.077, SRMR = 0.048) all meet commonly accepted standards (*χ*^2^/*df* < 3, CFI/TLI > 0.9, RMSEA/SRMR<0.08), indicating overall good model fit. The NFI = 0.89 and GFI = 0.844 are slightly below the ideal standard of 0.90, potentially due to the relatively low sample size relative to the number of observed variables. However, considering the strong performance of the core fit indices and the model’s solid theoretical foundation, this level of fit remains acceptable. Specific data are shown in Table 4: Model Fit Indices.

**Table 4:** Goodness of fit index of the structural model.

Fit index	*χ*^2^/*df*	CFI	NFI	TLI	GFI	RMSEA	SRMR
Suggested value	0–3	>0.900	>0.900	>0.900	>0.900	<0.08	≤0.08
Value of this study	2.552	0.931	0.891	0.920	0.844	0.077	0.048

## Discussion

This study focuses on the intensive care unit (ICU)—a high-stress, high-risk specialized nursing setting—and thoroughly examines the parallel mediating effects of occupational burnout and job satisfaction on the relationship between nurses’ work stress and turnover intentions. It provides empirical support for nursing management theory while offering practical guidance for healthcare institutions developing ICU nurse retention strategies. The findings of this study clearly confirm that occupational burnout and job satisfaction play significant parallel mediating roles in the relationship between work stress and turnover intention among ICU nurses. Specifically, work stress increases nurses’ turnover intention by significantly elevating occupational burnout levels and reducing job satisfaction.

This study found that work stress is significantly and positively associated with turnover intentions among intensive care unit nurses. This finding aligns with previous research by Zhang et al. (25) and Said and El-Shafei (46), who identified a direct relationship between work stress and nurses’ desire to leave their positions. This indicates that work stress itself is a major factor driving nurses to consider leaving, likely because sustained high-pressure work leads to physical and mental exhaustion, making it difficult for nurses to persist in their current roles. However, the effect size for ICU nurses is significantly higher (while the direct correlation coefficient between stress and turnover intention for general ward nurses typically ranges between 0.4 and 0.6, this study reached 0.758). This disparity underscores the unique stressors inherent to the ICU environment: Compared to general wards, ICU nurses face intense psychological impact from three primary pressures—life-responsibility stress (where procedural errors may directly result in patient death), 24/7 monitoring stress (requiring constant vigilance over critically ill patients’ vital signs), and ethical dilemma stress (such as resuscitation decisions for terminally ill patients). These factors translate stress into resignation intent more directly and rapidly. From the JD-R theoretical perspective, this direct effect essentially stems from the severe imbalance between the extreme demands of intensive care unit work (high risk, high workload) and limited work resources (understaffing, lack of psychological support). This imbalance has surpassed the indirect influence of health impairment pathways, directly triggering nurses’ tendency to make decisions to leave their jobs.

From the perspective of the burnout pathway, ICU nurses’ burnout is positively correlated with their intention to leave. Wang et al. (47) confirmed in their cross-sectional study that ICU nurses’ burnout is strongly correlated with turnover intention, consistent with the findings of this study, consistent with previous research findings (48, 49). Research indicates that the work stress experienced by ICU nurses directly induces occupational burnout. This burnout, in turn, undermines nurses’ mental health, leading to emotional exhaustion that diminishes their passion for work, dehumanization that fosters indifference, and reduced personal accomplishment. Ultimately, these factors drive nurses toward the intention to resign. This aligns with the core tenet of Maslach’s burnout theory that prolonged occupational stress is the primary cause of burnout, which in turn leads to individual avoidance behaviors such as job turnover.

From the perspective of job satisfaction pathways, research indicates that work stress significantly reduces ICU nurses’ job satisfaction, while low job satisfaction further intensifies their intention to leave. This finding aligns with Herzberg’s Two-Factor Theory, which posits that “work stressors (such as overload or excessive risk) constitute hygiene factors; their prolonged presence directly diminishes employee satisfaction and subsequently triggers turnover tendencies. Research findings indicate that work stress significantly impacts job satisfaction. This may stem from ICU nurses requiring greater time and effort to master specialized skills (such as ventilator operation and blood purification techniques), with each procedure directly impacting patient safety. This “high-cost, high-risk” nature of the work intensifies the corrosive effect of stress on satisfaction. When nurses endure prolonged high-intensity stress without receiving corresponding psychological compensation (such as the sense of accomplishment from patient recovery or organizational support), negative perceptions of their work accumulate more rapidly, thereby accelerating the increase in turnover intentions.

Based on the research findings, healthcare institutions can adopt a multi-pronged approach to reduce nurses’ intention to leave. Interventions for Professional Burnout: Results indicate that emotional exhaustion scored highest among burnout dimensions, forming the core mediating pathway. Specific recommendations include: providing ICU nurses with regular psychological counseling (e.g., monthly group therapy sessions) and establishing emotional support groups; optimizing shift schedules to avoid consecutive night shifts exceeding 3 days, ensuring adequate physiological rest to alleviate emotional exhaustion. To enhance job satisfaction: The “career development” dimension scored lowest in job satisfaction, followed by the “compensation and benefits” dimension. Specific recommendations include: establishing specialized career development pathways for ICU nurses (e.g., critical care specialist certification programs), providing targeted training and promotion opportunities; optimizing compensation structures by introducing critical care allowances and high-risk position subsidies to improve salary satisfaction. Addressing root causes of work stress: The dimensions “patient care issues” and “time allocation and workload” scored highest in work stress. Specific recommendations include: Rationally allocate human resources, maintaining a nurse-to-patient ratio within 1:2; optimize nursing processes by introducing intelligent care equipment (e.g., remote monitoring systems) to reduce repetitive tasks and alleviate operational pressure.

### Limitations and future research directions

First, the sample used in this study may have certain limitations. First, Convenience sampling may result in insufficient sample representativeness and introduce selection bias (e.g., exclusion of ICU nurses from primary-level hospitals). Future studies may adopt multistage stratified sampling to enhance sample representativeness. Second, this study employs a cross-sectional design, which can only reveal correlations between variables without establishing their causal sequence. Cross-sectional data cannot determine the temporal sequence between variables, presenting certain limitations in inferring mediating effects. Third, in terms of variable measurement, the study primarily relies on scale-based assessments. Self-report scales inherently carry subjective biases. When completing these scales, nurses may experience discrepancies between their evaluations of variables and actual circumstances due to factors such as social desirability and memory bias, thereby affecting the accuracy of research findings. Finally, the study focused solely on job satisfaction and burnout as mediating variables. In reality, other factors—such as organizational support, family factors, and personal personality traits—may also mediate or moderate the relationship between work stress and nurses’ turnover intentions. These variables, excluded from the research, could influence the findings, potentially leading to an incomplete explanation of the mechanisms through which work stress affects nurses’ turnover intentions.

To advance current knowledge in this field, future research should explore multiple avenues. First, employing longitudinal designs will help understand the long-term impact of work-related factors on turnover intentions and reveal causal mechanisms. Second, incorporating broader variables such as organizational culture, social support, job autonomy, and other relevant psychosocial factors will contribute to a more comprehensive understanding of the complexity involved. Furthermore, expanding the scope of investigations to encompass diverse healthcare settings both domestically and internationally will enhance the applicability and external validity of research findings. Additionally, qualitative research methods such as interviews or focus groups can be employed to gain deeper insights into nurses’ experiences and perspectives regarding work-related factors, burnout, and turnover intentions. These qualitative approaches will complement quantitative findings and provide a more profound understanding of underlying mechanisms.

## Conclusion

Through correlation analysis and structural equation modeling validation, this study concludes that significant correlations exist between job stress, job satisfaction, occupational burnout, and nurses’ turnover intentions. Job stress exerts a significant direct effect on nurses’ turnover intentions, while job satisfaction and occupational burnout play crucial parallel mediating roles in the process by which job stress influences turnover intentions. These findings provide scientific evidence for developing ICU nurse retention strategies in healthcare institutions and offer new empirical support for research on the “stress-to-turnover” mechanism within nursing management.

## Data Availability

The original contributions presented in the study are included in the article/supplementary material, further inquiries can be directed to the corresponding author.
